# Enhancing Food Production by Sustainable Cricket Farming in Thailand: Evaluating Black Soldier Fly Larvae as a Cost-Effective Feed Ingredient

**DOI:** 10.3390/insects16080856

**Published:** 2025-08-18

**Authors:** Sopa Cansee, Siripuk Suraporn, Nuntawat Butwong

**Affiliations:** 1 Faculty of Engineering, Mahasarakham University, Maha Sarakham 44150, Thailand; nuntawat.b@msu.ac.th; 2 Department of Biology, Faculty of Science, Mahasarakham University, Maha Sarakham 44150, Thailand; siripuk.s@msu.ac.th

**Keywords:** *Acheta domesticus*, *Hermetia illucens*, feed formulation, feed conversion ratio (*FCR*), alternative protein, nutrient efficiency

## Abstract

The high cost of commercial feed presents a major barrier to scaling environmentally responsible cricket (*Acheta domesticus*) farming, particularly in low-resource settings. We investigated the use of *Hermetia illucens* (Black Soldier Fly Larvae, BSFL) combined with locally available ingredients to develop affordable, nutritionally balanced feed for *A. domesticus*. A three-phase experimental approach was used: (i) feed preference assessment from six ingredients; (ii) performance evaluation of four BSFL-to-commercial feed ratios; and (iii) formulation and testing of three practical recipes using Pearson’s square method. Feed efficiency was measured via feed conversion ratio (*FCR*), efficiency of conversion of ingested food (*ECI*), and yield. A recipe consisting of 20.6% BSFL, 14.6% soybean meal, 29.1% corn meal, 17.8% rice meal, and 17.8% commercial feed achieved the best performance with an *FCR* of 2.2, *ECI* of 44.4%, and juvenile larvae yield of 99.9%. This formulation also reduced feed cost by 33% compared to commercial feed. The study confirmed that integrating BSFL with regionally sourced materials using a simple feed design method can enhance food security, reduce input costs, and support the transition toward circular insect farming systems.

## Simple Summary

Cricket farming is emerging as a sustainable protein source, but high feed costs limit its viability—especially for small farmers in tropical regions. This study developed an affordable, nutritionally adequate cricket feed source by incorporating *Hermetia illucens* (Black Soldier Fly Larvae, BSFL) and local agricultural by-products. Through a structured evaluation involving feed preference tests, BSFL blending ratios, and practical recipe formulation using Pearson’s square method, we determined an optimized diet that reduced feed costs by over 33%, while maintaining high growth performance. We presented feasible solutions for decentralized, low-cost cricket production that contribute to resource-efficient food systems in resource-limited areas.

## 1. Introduction

Global demand for sustainable protein sources continues to grow due to population expansion, environmental concerns, and the need for resilient food systems [1,2,3]. Conventional livestock production, while meeting current protein needs, remains resource-intensive, requiring significant land, water and feed inputs, and contributes substantially to greenhouse gas emissions [4,5,6,7,8]. Thus, edible insects have emerged as a promising alternative protein source, recognized by the Food and Agriculture Organization (FAO) for their environmental efficiency and nutritional quality [9,10]. Commercially reared species of crickets from the order *Orthoptera* and family *Gryllidae* are increasingly recognized for their favorable nutritional profile, including protein content comparable to that of traditional meat and fish, along with balanced amino acids, vitamins, and minerals [11,12].

However, one of the primary barriers to scalable cricket production is the high cost of feed, which may account for 50–60% of total production expenses [13,14]. While commercial feeds provide consistent nutritional quality, they are often prohibitively expensive for small farmers in developing countries. This has prompted exploration into alternative feed strategies, using locally available ingredients, including cereal by-products, agricultural waste, or insect-derived proteins [15,16]. Among these, *Hermetia illucens* (Black Soldier Fly Larvae, BSFL) have attracted significant attention due to their high protein (up to 45%) and fat content, ability to convert organic waste into biomass, and adaptability to various farming conditions [17,18,19]. Despite these advantages, knowledge gaps remain regarding the optimal BSFL inclusion levels in cricket feed, particularly in terms of palatability, feed conversion, and cost-efficiency.

A major challenge in the formulation of insect diets is achieving a balance between nutritional adequacy and ingredient accessibility, especially in low-resource contexts. While many commercial feeds are designed with complex nutritional software, small farmers require simple, low-cost tools to design effective rations. The Pearson’s square method is a classical feed formulation technique used to calculate ingredient ratios based on known nutrient values, typically proteins [20]. Although well-established in livestock nutrition, its application in insect farming remains limited, despite its ability to optimize economically viable, nutritionally balanced diets [21,22]. Integrating BSFL and other locally sourced ingredients into feed formulations using Pearson’s method offers a practical approach to reducing feed costs, while maintaining cricket performance.

Therefore, this study aimed to form and evaluate affordable cricket feeds by incorporating BSFL with locally available agricultural ingredients. It specifically addressed four objectives: (i) to assess the feed ingredient preference of *A. domesticus*, (ii) to evaluate feed conversion efficiency and growth performance under varying BSFL-to-commercial feed ratios, (iii) to form optimal feed recipes using the Pearson’s square method, and (iv) to analyze feed efficiency, biomass yield and optimal cost. By integrating biological performance with economic feasibility, this study contributes locally adaptable feed solutions for small-scale cricket farming.

## 2. Materials and Methods

### 2.1. Ingredient Materials and Cost

This study focused on developing an economically viable cricket feed using locally available ingredients to substitute for high-cost commercial feed. For application by small farmers, selected ingredients must be nutritionally adequate, economically viable, readily accessible, and environmentally responsible over the long term. Key ingredients included BSFL, soybean meal, corn meal, rice meal, and fermented cassava pulp. BSFL are highly nutritious and widely recognized as a sustainable protein source in animal husbandry. In many Thai communities, BSFL are reared using household or restaurant organic waste, often supported by local initiatives from the Department of Livestock Development. Farmers may raise BSFL independently or acquire larvae from regional suppliers. In this study, the cost of BSFL was estimated at 10 THB/kg, based solely on the energy cost of infrared drying [23,24,25]. This estimate was derived from internal pilot-scale drying trials using infrared equipment in the same research project. While related work by Butwong et al. [25] provided specific energy consumption (SEC) and drying parameters, the cost figure used here was independently calculated and reflects energy expenditure only. It did not include labor, equipment depreciation, or packaging costs, which may vary depending on farm practice and drying method.

Soybean meal and corn meal were obtained from the Maha Sarakham Dairy Farmers Cooperative Limited (16°16′20″ N, 103°17′10″ E), which supports 106 member farmers with over 4700 dairy cows. This cooperative has participated in government-supported modernization projects since 2017. Soybean meal and corn meal are commonly used in dairy cattle nutrition and were selected for their high nutrient density and availability. The costs were 18 THB/kg for soybean and 12 THB/kg for corn meal.

Rice meal is also widely accessible in rural Thai households, where rice is cultivated. Though commercial rice retails at about 20 THB/kg depending on variety and quality, here locally sourced rice meal was estimated at 10 THB/kg. Fermented cassava pulp, a by-product of cassava starch production, is often used in ruminant diets to reduce feed costs. While its raw market price is approximately 5 THB/kg, the cost increased to 8 THB/kg after drying and grinding to make it suitable for cricket feed. Fermentation enhances its nutritional value and digestibility [26].

Cereal-based ingredients—soybean meal, corn meal, and fermented cassava pulp were—obtained from local feed suppliers, while BSFL and rice meal were sourced from regional producers. Upon receipt, BSFL were inspected for uniformity and vitality following Sheppard et al. [27]. The BSFL cost estimate—10 THB/kg—was based on pilot-scale infrared drying trials and reflects only the energy consumption, calculated using SEC values. Labor, equipment depreciation, and packaging were excluded due to their high variability across regions and production systems. This cost estimate aligns with smallholder conditions in rural Thailand, where on-farm or community-level production is common. Therefore, the costs are illustrative and context-dependent rather than universally applicable.

Table 1 presents the nutrient composition of these ingredients, drawn from literature sources, to demonstrate their suitability in cricket feed formulations. It is important to note that actual nutrient values may vary depending on production methods, source material, and processing quality. However, the reported ranges reflect sufficient nutritional quality to support cricket growth in local farming contexts.

The protein content among the selected ingredients varied from 8% to 45%, while fat content ranged from 0.86% to 6.6%. Cricket feed generally targets a protein level of at least 21%, similar to commercial benchmarks, to meet the rapid growth requirements of *A. domesticus* due to their short life cycles. However, over-supplementation with protein can elevate feed costs. Jamjanya and Thavornanukulkit [31] suggested that 15% protein may be sufficient for optimal cricket development under field-adapted conditions. Prices were calculated based on prevailing local market conditions in northeastern Thailand (March 2024). While some variation in cost may occur seasonally or by region—especially for imported or feed-grade ingredients like soybean meal—ingredients such as rice meal and BSFL are widely accessible through local farming systems. This enhances feed price stability and supports decentralized, farmer-managed production. All feed ingredients were ground using a laboratory-grade grinder and passed through a No. 40 mesh sieve to ensure uniform particle size for improved feed intake and mixing consistency [29].

### 2.2. Cricket Farming Setup and Sampling

Cricket farming experiments were conducted using custom-built gypsum board enclosures measuring 1.2 m in width, 2.4 m in length, and 0.6 m in height. Nylon mesh netting covered the top of each enclosure to prevent cricket escape and to exclude common predators such as birds, lizards, and geckos. Internally, egg tray paper was used to provide vertical surface area and shelter to support molting behavior and reduce cannibalism—an issue frequently reported during the vulnerable molting phase [10,32].

Each production cycle lasted between 40–45 days and yielded approximately 10–15 kg of *A. domesticus*, depending on variables such as stocking density, feed regime, and environmental conditions. Cricket farming also generates substantial amounts of waste in the form of frass and uneaten feed. Prior research suggests that total waste generation may range from 1.0–2.0 times the live weight of harvested crickets [33,34]. Environmental variables such as temperature and humidity strongly influence cricket growth, feed conversion, and molting frequency. Optimal production is typically achieved under conditions of 30–35 °C and 60–75% relative humidity [35].

Commercial cricket feed was administered twice daily, typically between 6:00–9:00 a.m. and 4:00–6:00 p.m., although actual timing was flexible depending on farm logistics. Feeding frequency was critical to prevent starvation and feed amounts were adjusted based on age and density to avoid waste accumulation and ammonia buildup. Maintaining sanitation and aligning feed amounts with physiological demands are necessary to safeguard health and microbial safety in rearing systems [33].

Water was provided ad libitum using shallow plastic trays placed inside the rearing units. Adequate hydration was especially important during molting stages, when dehydration could compromise exoskeleton hardening [10,36].

The species selected for this study was *A. domesticus*, which is often co-reared with *Gryllus bimaculatus* in Thai cricket farms. To ensure species purity, a three-generation purification method was used. Approximately 300–400 mature females were allowed to oviposit in containers lined with coconut husk and moist black rice husk. The eggs were then incubated and reared in the same enclosure design described above. Because first-generation populations may contain genetic admixture, subsequent rounds of selection were carried out until a pure *A. domesticus* population was obtained.

The experimental trials were conducted at the Sufficiency Economy Learning Center, Ban Don Man (16°16′17.41″ N, 103°12′43.55″ E) and in collaboration with a large-scale farm at Hong Hee Village. The trial period spanned March to April 2024, during which the average daily temperature ranged between 35–40 °C and the relative humidity varied from 69–72%, based on data from the Thai Meteorological Department [34].

### 2.3. Feed Testing Procedures

**Figure 1:**
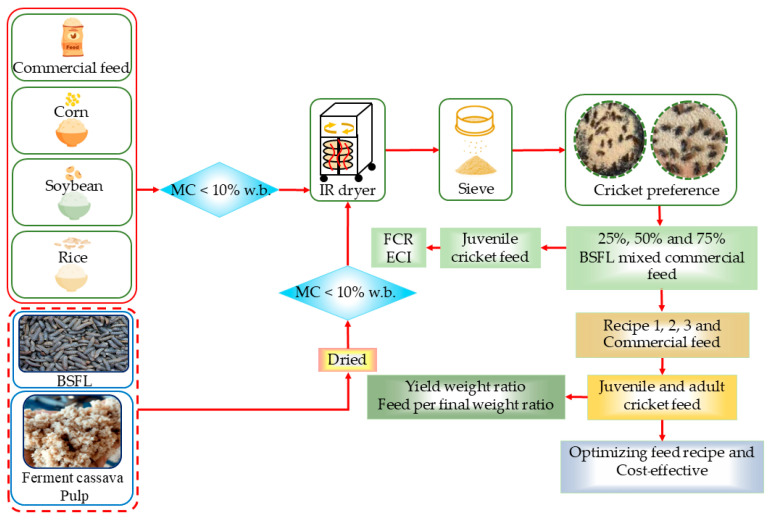
Schematic of the three phases of the experimental framework in this study: (1) ingredient preference assessment using 21-day-old *A. domesticus*; (2) evaluation of growth performance under varying BSFL-to-commercial feed ratios; and (3) formulation and validation of feasible feed recipes using Pearson’s square method. Each stage was conducted under simulated small-scale cricket farming conditions in gypsum rearing ponds.

Feed testing used 21-day-old *A. domesticus*, selected on age and average mass to ensure developmental uniformity and minimize handling stress during sampling. A structured three-phase experimental design, illustrated in Figure 1, was used: (1) feed ingredient preference assessment, (2) evaluation of BSFL-to-commercial feed blending ratios, and (3) formulation of experimental diets using Pearson’s square method. Each phase was designed to enhance feed efficiency and reduce costs while maintaining optimal biological performance in *A. domesticus*.

#### 2.3.1. Cricket Feed Preference Assessment

This initial phase followed established protocols for evaluating palatability in insect feed studies, using residual feed mass as a proxy for ingredient [37]. Ingredients with low palatability can lead to high feed residue, which not only increases waste but also contributes to pond ammonia buildup, creating a hazardous environment for crickets [38].

The ingredients evaluated—BSFL, soybean meal, corn meal, rice meal, and fermented cassava pulp—were ground into fine powders and sieved to ensure uniform particle size, which improves feed intake and digestion [39].

**Figure 2:**
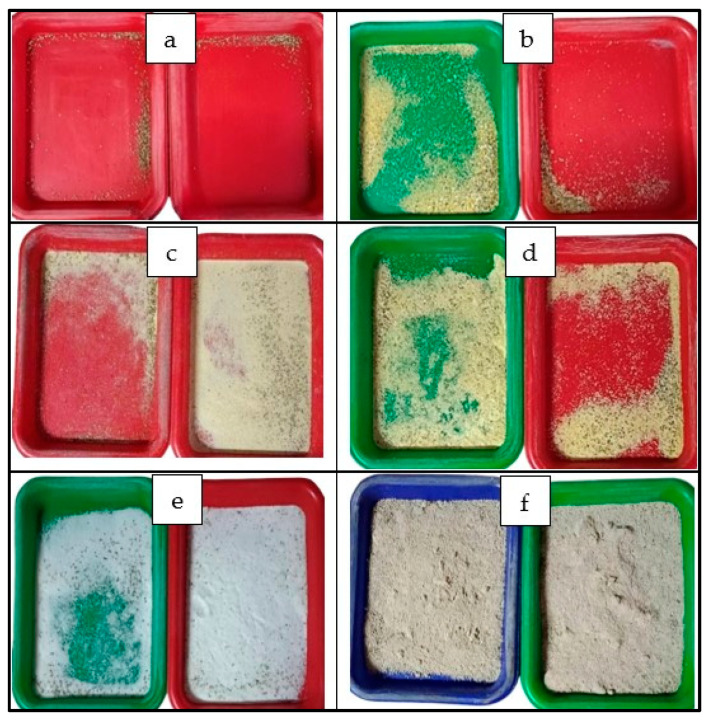
Visual appearance of residual feed from cricket preference trials for each ingredient after a 120-min feeding period. Lower residues indicate higher feed acceptability. Photos of two replicates per ingredient are shown: (**a**) BSFL powder; (**b**) Commercial feed; (**c**) Soybean meal; (**d**) Corn meal; (**e**) Rice meal; and (**f**) Fermented cassava pulp.

**Figure 3:**
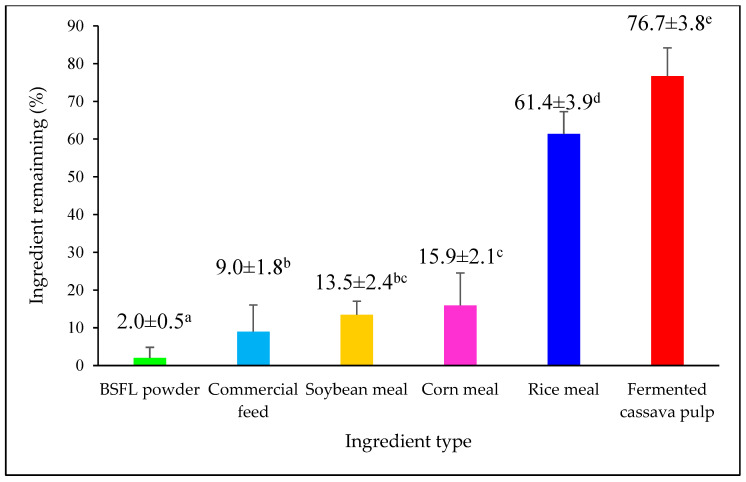
Fractions of remaining feed shown by crickets: BSFL, commercial feed, soybean meal, corn meal, rice meal, and fermented cassava pulp. Values are presented as mean ± standard deviation (SD). Different superscript letters indicate significant differences (*p* < 0.05), n = 2, one-way ANOVA with Tukey’s HSD.

Each ingredient (50 g) was placed in plastic feeding trays (16 × 20 × 2.5 cm), each containing 21-day-old *A. domesticus*. This stage was chosen based on known dietary sensitivity during early growth [29]. Two replicates per ingredient (12 trays total) were arranged randomly within a full-sized gypsum rearing enclosure to avoid positional bias. Feed volumes were adapted to the density of each ingredient to ensure tray capacity was standardized across treatments. Feed preference trials were conducted during the second daily feeding session (16:00–18:00), following a short fasting period. After 120 min, residual feed was collected and weighed to calculate the percentage of uneaten feed. Ingredients with the lowest residuals were prioritized for inclusion in later feed recipes, while those with high residuals—such as fermented cassava pulp were excluded due to their lower palatability and potential for contributing to feed spoilage [10]. Feed intake was visually observed (Figure 2) and the residual quantities of each ingredient were collected and weighed to quantify feed preference (Figure 3). Although only two replicates per treatment were used in this phase, this limited design was intended to reflect common constraints in smallholder systems and to serve as a feasible, indicative screening step prior to the more robustly replicated feed trials described in Section 2.3.2 and Section 2.3.3.

#### 2.3.2. BSFL and Commercial Feed Ratio Testing

In the second phase, four feed treatments were formulated by blending BSFL powder and commercial cricket feed in the following weight-to-weight (*w*/*w*) ratios: 25:75, 50:50, 75:25, and 0:100 (commercial feed only). These combinations were selected based on past studies exploring the integration of insect meal into poultry and insect diets [40]. Each formulation was standardized through drying and grinding to ensure consistency. A total of 500 g of 21-day-old *A. domesticus* were placed into 1 m^2^ gypsum board rearing units for each treatment. Crickets were fed twice daily and feed waste was monitored and removed to prevent ammonia accumulation, which has been associated with mortality and suppressed growth [29,34].

Cricket biomass and feed intake were measured over a 14-day period. At the conclusion of the trial, crickets were harvested and weighed for *FCR, ECI*, and biomass yield. Each treatment was replicated three times, ensuring statistical rigor in the evaluation of performance metrics.

#### 2.3.3. Pearson’s Square-Based Feed Formulation

In the final phase, experimental feed formulations were developed using Pearson’s square method, targeting a minimum crude protein level of 21% to match commercial feed standards [22,41]. Though originally a two-ingredient method, Pearson’s square served as a foundational tool in designing more complex, multi-ingredient formulations [18]. For example, Recipe 1 was initially formulated using BSFL powder (45% protein) and corn meal (8.04% protein), which yielded a base mixture of approximately 38.15% BSFL and 61.85% corn meal. To optimize the nutritional profile while lowering BSFL content, additional components—soybean meal (42.37% protein), commercial feed (21% protein), and rice meal (8%)—were added. Iterative recalculations adjusted the final proportions to: 15.80% BSFL, 19.98% soybean meal, 31.61% corn meal, 21.74% rice meal, and 10.87% commercial feed, yielding a crude protein content of 22.14%.

Ingredient selection was based on functional roles: BSFL and soybean meal provided high-quality protein; corn and rice meal supplied carbohydrates and texture; and commercial feed offered baseline micronutrients. The protein level achieved aligns with nutritional needs for both juvenile and adult *A. domesticus* [36,42].

An economic analysis based on local market prices (THB/kg) was conducted: BSFL (10 THB), soybean meal (18 THB), corn meal (12 THB), rice meal (10 THB), and commercial feed (21 THB). The estimated cost for Recipe 1 was 13.32 THB/kg, representing a 36.57% reduction compared to the use of commercial feed alone. These results affirm the economic feasibility and biological efficacy of BSFL-based feed as a resource-efficient alternative for small-scale cricket farming [16,19,20].

### 2.4. Practical Cricket Feed Recipe Development

Based on the results of ingredient preference testing and BSFL inclusion ratio assessments, three practical feed formulations were developed using Pearson’s square method, ensuring that each blend met a minimum crude protein level of 21%, consistent with commercial cricket feed standards [17,27]. The formulations incorporated various proportions of BSFL, soybean meal, corn meal, rice meal, and a commercial control feed. These combinations were strategically designed to achieve nutritional adequacy, ingredient synergy, and low cost, targeting both protein content and local ingredient accessibility. 

To evaluate the biological and economic viability of the developed formulations, feeding trials were conducted using *A. domesticus* at two developmental stages: (i) 900 g of juvenile crickets (~21 days old) and (ii) 500 g of mature adults. This stage-based approach allowed assessment of feed performance under varied physiological demands—juveniles typically require higher protein for growth and tissue development, while adults nearing the end of their lifecycle may perform adequately with lower-protein diets supplemented with fibrous material [9,10].

All trials were conducted under ambient environmental conditions that reflect typical small-scale cricket farming systems in Thailand, with temperature and humidity aligned with seasonal norms [34,43]. Crickets were reared in 1 m^2^ enclosures to ensure uniform stocking density. Feed was administered twice daily, and water was provided ad libitum through shallow trays. Each formulation was tested in triplicate for statistical reliability. Performance indicators included feed intake, *FCR*, and growth yield, benchmarked against a commercial control diet. The aim was to identify the most efficient, financially accessible, and field-adapted feed formula that could support continuous production in resource-limited settings [44].

### 2.5. Performance Indicators

Cricket production involves large-scale rearing, during which crickets undergo multiple molts. The molting renders them physiologically vulnerable, often leading to temporary weight loss and increased susceptibility to cannibalism [45]. Consequently, final body mass may increase, decrease, or remain unchanged relative to initial body mass, even though overall size typically increases during development.

To assess the nutritional performance of each feed treatment, we used three key quantitative indicators:

Feed Conversion Ratio (*FCR*) measures the efficiency with which crickets convert feed into body mass. It is defined as the mass of feed consumed per unit of mass gained during the experimental period:

(1) $$FCR=\;\frac{Feed\;given}{Animal\;weight\;gain}$$

In essence, *FCR* is the relative mass gain of the crickets: it is commonly used in insect farming to assess feed efficiency [46].

Efficiency of Conversion of Ingested Food (*ECI*) measures the proportion of ingested feed successfully converted into body mass, expressed as a percentage:

(2) $$ECI=\;\frac{Weight\;gained}{Feed\;intake}\times \;100$$

This indicator reflects nutrient assimilation efficiency, which is critical in evaluating alternative feeds like BSFL [47]. High *ECI* values indicate better conversion of feed nutrients into cricket biomass.

Yield (*W_y_*) represents the final mass of crickets as a percentage of their initial mass, capturing net growth relative to initial biomass:

(3) $$W_{y}=\;\frac{W_{f}}{W_{i}}\times 100$$

where *W_f_* is the final mass of the cricket population (g), and *W_i_* is the initial mass of the cricket population (g). This indicator was particularly useful for comparing feed efficiency across juvenile and adult cricket stages [48].

### 2.6. Data Analysis

Experimental data from the feed preference test, BSFL ratio trials, and practical recipe evaluations were analyzed using one-way analysis of variance (ANOVA) to determine significant differences among treatments. Unless otherwise specified, statistical tests used the conventional *p* < 0.05 criterion, implying less than 5% probability that the result was the result of pure chance. All analyses used IBM SPSS Statistics Version 29.0.2.0 (Released 2023, IBM Corp., Armonk, NY, USA). Tukey’s Honestly Significant Difference (HSD) test was used for post hoc comparisons. Prior to applying ANOVA, key assumptions were verified: normality of residuals was assessed using the Shapiro–Wilk test and homogeneity of variances was evaluated using Levene’s test. In all cases, assumptions were met (*p* > 0.05), supporting the use of parametric methods. The 75% BSFL inclusion group was excluded from the statistical analysis of *FCR* and *ECI* due to negative mass gain, which rendered these metrics non-computable. Nevertheless, its outcome was discussed in the results section to highlight the implications of excessive BSFL inclusion.

## 3. Results

### 3.1. Ingredient Feed Preference

The feeding behavior of *A. domesticus* was evaluated over a 120-min period to assess the palatability of individual feed ingredients. BSFL powder showed the highest acceptability, with only 2.0 ± 0.5% residual feed remaining. Commercial feed followed closely, with 9.0 ± 1.8% remaining feed. Both these ingredients were significantly more consumed than others, indicating their suitability as primary components in cricket diets.

Soybean meal and corn meal were moderately acceptable, with residuals of 13 ± 2% and 16 ± 2%, respectively, but statistically there was no significant difference. Although not as preferred as BSFL or commercial feed, both soybean and corn meal remained within the acceptable range for inclusion in feed formulations.

In contrast, rice meal and fermented cassava pulp were little consumed, leaving high residuals of 61 ± 4% and 77 ± 4%, respectively. This suggests limited palatability and potential issues with texture, odor, or digestibility. Therefore, fermented cassava pulp was excluded from further formulations due to its low acceptance and preparation complexity. While rice meal performed poorly, it was retained in recipes due to its affordability, ease of access, and familiarity among small farmers. When combined with higher-quality protein ingredients, rice meal adds an energy contribution and feed structure in mixed formulations.

These results emphasized the importance of selecting ingredients that balance palatability, nutritional adequacy, and practical feasibility; thus, BSFL, commercial feed, soybean meal, corn meal, and rice meal were selected for subsequent trials.

### 3.2. BSFL and Commercial Feed Combination

Substantial differences in feed use metrics were observed across the diets (Table 2). Crickets fed the control diet (0% BSFL; 100% commercial feed) achieved a final weight of 820 g, yielding a *FCR* of 3.1 ± 1.3 and an *ECI* of 32.3 ± 5.4%. These baseline values reflected typical performance associated with conventional feeds in tropical farming contexts. Partial replacement of commercial feed with BSFL improved feed efficiency markedly. The 50% BSFL group recorded an *FCR* of 2.2 ± 0.2, significantly lower than the control, indicating greater efficiency in converting feed into biomass. Similarly, this group showed the highest *ECI* (44.4 ± 6.3%), suggesting superior nutrient assimilation and metabolic efficiency. These improvements were attributed to the balanced nutrient synergy between animal-derived BSFL protein and plant-based feed ingredients. The 25% BSFL group showed moderate performance, with an *FCR* of 3.7 ± 0.5 and *ECI* of 27.3 ± 2.4%. However, these values were not statistically different from the control group, indicating that low inclusion rates did not provide sufficient nutritional enhancement to improve feed efficiency.

Conversely, the 75% BSFL group resulted in poor performance, with a final mass of 461 g—lower than the initial biomass—indicating negative growth. Therefore, *FCR* and *ECI* could not be calculated. This performance decline was attributed to excessive BSFL content, which may have led to nutritional imbalances, particularly in carbohydrate limitation, and increased intake of indigestible chitin or excess lipid, impaired feed digestibility, and satiety regulation.

Overall, moderate inclusion of BSFL at 50% of the diet yielded the most favorable performance in terms of both feed efficiency and mass gain. These results support the potential of BSFL as a high-performing ingredient in cricket feed when incorporated at appropriate ratios.

### 3.3. Ingredient Ratio Recipes

Feed formulation using Pearson’s Square method yielded three experimental diets incorporating BSFL, soybean meal, corn meal, and rice meal (Table 3). Each formulation was designed to meet a minimum crude protein target of 21%, in line with nutritional standards for commercial cricket feeds. BSFL inclusion levels were for Recipe 1—15.8%, Recipe 2—20.6%, and Recipe 3—22.6%. Notably, Recipe 3 was formulated entirely without commercial feed, demonstrating the potential for fully self-sufficient feed strategies.

Feed quality during storage was an important consideration, especially under warm and humid tropical conditions common to small cricket farms. All formulations had moisture contents below the recommended 13% wet basis (w.b.) threshold for microbial stability, with values of 10.1%, 8.2%, and 8.2% for Recipes 1, 2, and 3, respectively. These levels were suitable for medium-term storage with minimal risk of spoilage.

Economically, all three experimental recipes outperformed the commercial control (Recipe 4), at 21 THB/kg. The cost per kilogram for Recipes 1, 2, and 3 was 13.3, 14.2, and 12.4 THB, respectively. These figures correspond to cost reductions of 37%, 33%, and 41%, respectively. These savings highlighted the economic advantage of formulating feeds with regionally available ingredients while maintaining nutritional adequacy.

The Pearson’s Square method provided a flexible and accessible arithmetic approach to blend feed ingredients. While originally designed for two-ingredient formulations, the method was adapted here for multi-ingredient recipes through iterative calculations. Protein-rich components like BSFL and soybean meal were proportionally balanced with carbohydrate-rich ingredients such as corn and rice meal, yielding a complete feed suitable for supporting both growth and reproductive performance in *A. domesticus.*

Recipes 1 and 2 included a proportion of commercial feed, which helped maintain alignment with standard feed profiles and may assist farmers transitioning from full commercial diets. Recipe 3, which excluded commercial feed entirely, while still meeting protein and quality targets, demonstrated that regional feed production using local inputs was both feasible and effective.

Collectively, the results from moisture analysis, ingredient ratios, and cost calculations supported the conclusion that locally formulated feeds can be effective alternatives to commercial feeds. These findings support the development of locally adaptable, affordable, and scalable solutions for insect farming in low-resource environments.

### 3.4. Performance of Crickets Fed Different Recipes

**Figure 4:**
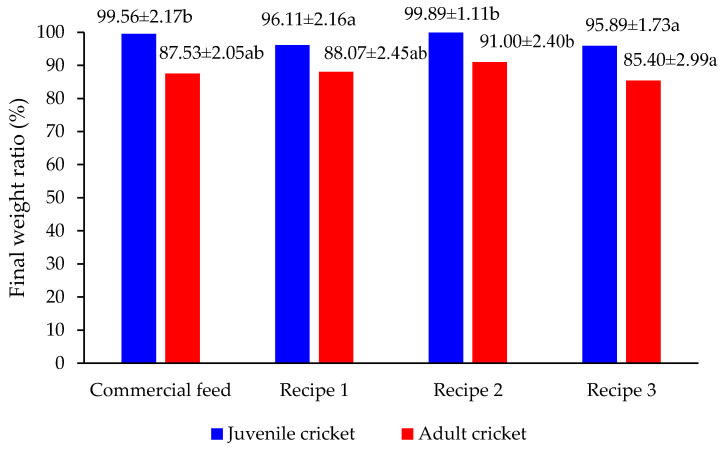
Yield ratio (%) of juvenile and adult *A. domesticus* fed different recipes. Values are presented as mean ± standard deviation (SD). Different letters (a, b) indicated significant differences, n = 3, one-way ANOVA with Tukey’s HSD.

Feeding trials evaluated the growth performance and feed efficiency of *A. domesticus* using three experimental diets versus a commercial control. As shown in Figure 4, juvenile crickets fed Recipe 2 achieved the highest yield-to-initial-mass ratio at 99.9 ± 1.2%, marginally surpassing the commercial feed (99.6 ± 1.3%) and significantly outperforming Recipes 1 (96.1 ± 1.5%) and 3 (95.9 ± 1.6%). All formulations were designed to meet or exceed the general 21% CP benchmark for *A. domesticus*, with Recipe 1 estimated at 22.1% based on known ingredient composition. However, ISO 12099 NIRS analysis revealed that Recipe 2 performance was attributed to its optimized nutrient profile, which included approximately 24.9% crude protein, 8.3% fat, 4.5% fiber, 26.2% starch, and 6.5% ash—levels that meet the metabolic demands for rapid growth and exoskeleton development in juvenile crickets.

In comparison, Recipes 1 and 3 yielded lower performance, due to their lower protein contents and less favorable fat-to-fiber ratios. Recipe 3, which excluded commercial feed entirely, included a higher proportion of rice meal, which may have reduced digestibility and nutrient absorption.

Adult crickets showed lower yields than juveniles across all diets, which is consistent with their reduced growth rates, metabolic activity, and feed consumption during maturity. High ambient temperatures during the trial period may have also contributed to suppressed feed intake and slower mass gain. Despite this, Recipe 2 continued to perform best for adult crickets, producing a 91% yield, compared to 87% for the commercial feed.

**Figure 5:**
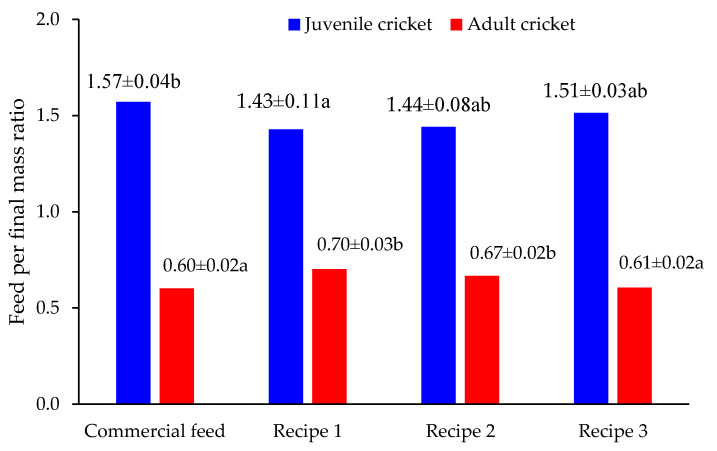
Feed per final mass ratio for juvenile (blue bars) and adult (red bars) *A. domesticus* versus different feed recipes. Values are presented as mean ± standard deviation (SD). Different letters (a, b) indicated significant differences, n = 3, one-way ANOVA with Tukey’s HSD.

Feed intake relative to mass gain was analyzed to assess feed conversion efficiency. As presented in Figure 5, juvenile crickets consumed between 1.43–1.57 g of feed per gram of body mass gained. Recipe 2 led to the most efficient feed conversion at 1.44 ± 0.06 g/g, significantly better than the commercial feed group at 1.57 ± 0.08 g/g. In adult crickets, feed intake per unit of mass gain was substantially lower, ranging between 0.60–0.70 g/g, reflecting physiological plateauing in growth.

These results validated Recipe 2 as both a nutritionally sufficient and economically favorable alternative to commercial feed, particularly for juvenile crickets, where protein acquisition is essential for rapid growth. The inclusion of BSFL and soybean meal contributed to its high-quality protein profile, while the moderate fat content enhanced palatability and energy availability. These findings are consistent with previous studies indicating that balanced macronutrient composition improved nutrient assimilation and reduced metabolic stress in crickets. Notably, Recipe 2 achieved the best balance between cost and performance: despite costing 33% less than commercial feed, it yielded significantly better *FCR* (2.31), *ECI* (42.8%), and final biomass across both juvenile and adult stages. This clearly demonstrated that cost reductions achieved through the use of locally sourced ingredients did not compromise, but rather enhanced production efficiency. This reinforced the real-world viability of adopting BSFL-based diets for locally adaptable cricket farming.

In summary, Recipe 2 offered a compelling feed formulation that combined high biological efficacy with affordability, supporting its application in resource-limited settings and contributing to the broader goals of environmentally responsible agriculture and resource-recycling protein systems.

## 4. Discussion

This study examined the biological performance and feed efficiency of cricket diets incorporating BSFL with a focus on palatability, nutritional adequacy, and field-adapted viability for small farms. Results demonstrated that BSFL inclusion substantially improved feed performance when blended with regionally available ingredients such as soybean, corn, and rice meals.

Palatability played a central role in feed intake, directly affecting growth in *A. domesticus*. BSFL powder was the most preferred, leaving only 2.0 ± 0.5% residual feed, followed by commercial feed (9.0 ± 1.8%). In contrast, rice meal and fermented cassava pulp were poorly accepted, leaving 61.4 ± 3.9% and 76.7 ± 3.8% residues, respectively. These findings align with prior work highlighting the importance of taste and texture in insect feed acceptance [49,50]. Crickets consuming highly palatable feeds tend to exhibit greater growth rates due to increased intake of essential nutrients, while low-palatable diets result in undernourishment and developmental delays [15]. It should be noted that the feed preference assessment was conducted with only two replicates per ingredient, primarily to reflect feasible constraints commonly encountered in smallholder cricket farming systems. This limited replication was intended as a preliminary, indicative screening to identify the most and least palatable ingredients for further formulation trials. Subsequent experimental phases, including performance and efficiency evaluations, were conducted with increased replication to ensure statistical robustness.

Beyond intake, palatability influences nutrient utilization. Selective or reduced feeding caused by unpalatable components can impair digestion and limit nutrient assimilation. Thus, even nutritionally balanced diets can underperform if poorly consumed [9].

A notable finding was the superior feed conversion ratio (*FCR* = 2.2 ± 0.2) and efficiency of conversion of ingested food (*ECI* = 44.4 ± 6.3%) observed in the 50% BSFL blend, significantly outperforming the control (*FCR* = 3.1 ± 1.3; *ECI* = 32.3 ± 5.4%). These results support existing literature endorsing moderate BSFL inclusion in insect diets for improved production efficiency [27,51]. The improvement is attributed to nutrient complementarity. BSFL provides proteins and lipids, while commercial feed balances carbohydrates and micronutrients.

This synergistic formulation enhances not only intake but also nutrient absorption. For instance, BSFL-derived lipids facilitate the uptake of fat-soluble vitamins and their moderate chitin content may act as a prebiotic, enhancing gut health [52]. Together, these factors contribute to the improved *ECI* and support optimal growth.

However, the 75% BSFL group showed negative growth and uncalculable *FCR* and *ECI* values, indicating nutritional imbalance. Excessive BSFL may lead to deficits in carbohydrates or certain micronutrients, increasing metabolic stress [53]. Moreover, the high chitin content at this inclusion level may function as an anti-nutritional factor, obstructing enzymatic activity and reducing digestibility [54]. Reduced feed palatability at this level is also plausible, as high concentrations of insect protein can produce undesirable sensory characteristics [55].

In conclusion, while BSFL is a valuable feed component for *A. domesticus*, its inclusion must be carefully optimized. A 50:50 BSFL-commercial blend represents a balanced approach, maximizing performance and feed efficiency while minimizing risks associated with over-reliance on a single ingredient.

The optimal feed formulation, Recipe 2, demonstrated superior biological performance and economic efficiency, making it a practical option for small farmers of *A. domesticus* in Thailand. Developed using Pearson’s square method, this blend of 20.6% BSFL and 14.6% soybean meal not only met the nutritional requirements of crickets but also addressed economic and post-harvest challenges relevant to small-scale systems. Its crude protein content of 24.9% exceeds the ≥21% threshold required for promoting rapid juvenile growth [50], while also delivering an amino acid profile essential for exoskeleton development and overall health. The inclusion of BSFL and soybean meal illustrates a complementary protein strategy—BSFL contributing high-quality animal-derived protein and beneficial lipids, and soybean meal serving as a low-cost, plant-based counterpart [51]. The strategic combination enhances amino acid balance and nutrient density, supporting optimal growth, particularly during critical developmental stages [56]. This synergistic approach aligned with broader research advocating multi-source protein feeds to improve digestibility and nutrient bioavailability [22].

Economically, the feed formulation delivered substantial cost savings of 33% (14.2 THB/kg vs. 21.0 THB/kg for commercial feed). Feed cost is a major barrier in cricket farming [19] and reducing reliance on expensive, imported ingredients is vital for sectoral expansion. This affordable formulation, using locally accessible ingredients such as BSFL reared on organic waste, aligns with circular economy models and promotes localized food sovereignty [9]. It is important to note, however, that the cost comparisons in this study are illustrative and highly context-dependent. The BSFL cost used (10 THB/kg) was derived from pilot-scale infrared drying trials and included only the energy consumption, excluding labor, packaging, or equipment depreciation. These excluded factors vary widely and were omitted to avoid introducing speculative assumptions. The economic model assumes that small farmers may produce certain ingredients (e.g., BSFL, rice meal) on-farm using low-cost labor, while others (e.g., soybean and corn meal) may be purchased. As such, actual feed costs may fluctuate significantly based on region, resource availability, and input sources. Nonetheless, the formulation approach remains adaptable and cost savings become more pronounced when locally produced inputs are maximized. The use of Pearson’s square method ensures replicability, enabling scalable implementation in resource-constrained settings.

Beyond nutritional and economic considerations, the study emphasizes feed safety and longevity, particularly in tropical regions where high humidity promotes microbial contamination. Maintaining moisture content below 13% w.b. is crucial to inhibit mold and bacterial growth [57,58]. Recipe 2’s 8.25% moisture content not only ensures shelf stability but also minimizes post-harvest losses, a significant concern for smallholders lacking access to advanced storage facilities. Low moisture also preserves physical feed quality, preventing spoilage and maintaining nutrient integrity [52,59].

Biological performance data further affirm the superiority of Recipe 2. Juvenile crickets achieved a yield-to-initial-weight ratio of 99.89%, while adults reached a final yield of 91.00%, indicating consistent growth across life stages. This reflects not only adequate protein and lipid content but also optimal energy balance. Juveniles require high protein and fat for rapid tissue development, whereas adults need stable energy intake for maintenance and reproduction [50,60]. The reduced feed consumption per gram of body mass gained (1.44 g/g vs. 1.57 g/g in the control) highlights superior feed conversion efficiency, reinforcing the formulation’s economic and ecological value [29,61].

One particularly insightful result is the effective use of rice meal in Recipe 2. Despite its low palatability when offered alone, its inclusion at 17.86% in the blend contributed to carbohydrate provisioning—critical for energy metabolism. Excess protein, if not balanced by carbohydrates, may be catabolized inefficiently, leading to metabolic stress and nitrogenous waste accumulation [42]. As such, rice meal serves as a low-cost energy substrate, reducing the reliance on more expensive protein sources for energy. Furthermore, its granular structure may enhance feed texture, improve palatability, and minimize waste, aligning with previous recommendations [10,18,62,63].

This synergy among ingredients reflects a systems-based approach to feed design, in which ingredient interactions are prioritized over isolated nutrient values. The balance achieved in Recipe 2 between protein, fat, and carbohydrates represents an optimal metabolic substrate matrix, supporting both anabolic growth and energy-demanding behaviors. Such balanced formulations are particularly important in tropical and subtropical production systems, where temperature and humidity fluctuations can impact feed stability and intake behavior.

From a sustainability perspective, BSFL inclusion is a particularly important innovation. Rearing BSFL on organic waste streams offers a dual environmental benefit: reducing waste and producing high-quality protein. Numerous studies confirm that BSFL farming significantly reduces greenhouse gas emissions, land use, and water demand compared to traditional livestock feed production [51,56,64,65]. The feed formulation thus contributes to a waste-to-value bioeconomy model, turning agricultural or household waste into insect biomass—a strategy increasingly supported by international development frameworks [7,9]. Additionally, the study’s reference to solar or infrared drying methods for BSFL processing further supports its low-carbon profile, making it suitable for integration into decentralized rural production systems.

Furthermore, this study exemplifies how scientific feed formulation can serve as a driver for rural economic empowerment. By using local ingredients, simple processing methods, and structured formulation tools, farmers can reduce dependency on imported commercial feeds, retain value within communities, and improve the profitability of cricket farming enterprises. This aligns with broader goals of climate-smart agriculture and supports United Nations Sustainable Development Goals related to zero hunger, responsible consumption, and economic growth.

The empirical success of the optimized feed formulation holds implications beyond cricket production. The principles demonstrated—ingredient synergy, nutritional balance, cost optimization, moisture control, and sustainability—are broadly transferable to other insect species and alternative protein systems. As such, this study contributes to the growing field of entomological feed sciences and supports the global shift toward environmentally responsible, insect-based food and feed solutions.

In summary, Recipe 2 delivers an optimal combination of nutritional adequacy, feed efficiency, economic viability, and product stability. The recorded performance metrics—*FCR* of 2.2, *ECI* of 44.4%, cost savings exceeding 33%, and high yields across developmental stages—demonstrate its practical suitability for smallholder cricket farming. The structured, evidence-driven methodology employed here offers a scalable model for advancing insect farming technologies across diverse agroecological contexts.

## 5. Conclusions

This study demonstrated that partial substitution of commercial cricket feed with Black Soldier Fly Larvae (BSFL) can significantly enhance feed efficiency and reduce production costs in *A. domesticus* farming. Among the tested formulations, Recipe 2 (containing 20.6% BSFL and 14.6% soybean meal) yielded the highest growth performance, with a feed conversion ratio (*FCR*) of 2.2 ± 0.2 and efficiency of conversion of ingested food (*ECI*) of 44.4 ± 6.3%, outperforming both the commercial control and other experimental diets. This indicates a synergistic effect between BSFL and plant-based proteins.

Economically, this formulation reduced feed cost by 33% compared to the commercial feed, offering a practical solution for cost-sensitive, smallholder cricket farmers. The findings support the viability of using locally sourced BSFL as a core protein ingredient, particularly in rural Thai contexts where labor and input costs are minimized. However, excessive BSFL inclusion (75%) negatively affected growth and feed utilization, suggesting that inclusion ratios must be carefully optimized. While the cost model is context-specific, the methodological framework—including Pearson’s square formulation and performance benchmarking—can be adapted across regions with similar agroecological and economic conditions.

These results provide a robust foundation for promoting sustainable, low-cost cricket feed strategies and support broader efforts toward circular food systems and localized protein production.

## Figures and Tables

**Table 1:** Types of local feed ingredients, their nutrient composition, and market prices.

No.	Ingredient	Protein (%)	Fat (%)	Fiber (%)	Calcium (%)	Price (THB/kg)	Ref.
1	BSFL powder	45.0	6.60	7.0	6.00	10	[17,27]
2	Soybean meal	42.4	0.86	6.0	0.22	18	[28]
3	Corn meal	8.0	6.05	2.0	0.00	12	[9]
4	Rice meal	8.0	4.00	2.4	0.03	10	[29]
5	Commercial feed	21.0	5.00	3.0	13.10	21	[30]
6	Fermented cassava pulp	16.0	—	12.0	—	8	[26]

Note: Nutrient composition values are based on references as cited. Market prices (THB/kg) represent local feed costs collected in March 2024 from suppliers in northeastern Thailand. THB refers to Thai Baht; 1 USD ≈ 33 THB.

**Table 2:** Growth performance of *A. domesticus* after a 14-day feeding trial with three trial diets and a commercial control.

BSFL Ratio (%)	Feed Intake (g)	Final Mass (g)	Mass Gain (g)	*FCR* (±SD)	*ECI* (%) (±SD)
0	992	820	320	3.1 ± 1.3 ^a^	32.3 ± 5.4 ^a^
25	1121	806	306	3.7 ± 0.5 ^a^	27.3 ± 2.4 ^a^
50	1068	974	474	2.2 ± 0.2 ^b^	44.4 ± 6.3 ^b^
75	889	461	n.c.	n.c.	n.c.

Note: Data are presented as mean ± standard deviation (SD). Different superscript letters (a, b) indicate statistically significant differences among treatments, as determined by one-way ANOVA followed by Tukey’s HSD post-hoc test. n.c. = Not calculable due to the final cricket mass being lower than the initial mass, resulting in negative growth.

**Table 3:** Ingredient ratios for cricket feed recipes (100 kg total weight basis).

Feed Ratio (%)	Recipe 1	Recipe 2	Recipe 3	Recipe 4 *
BSFL powder	15.80	20.61	22.63	
Soybean meal	19.98	14.56	15.99	
Corn meal	31.61	29.12	31.97	
Rice meal	21.74	17.86	29.41	
Commercial feed	10.87	17.86	-	100.00
Total weight (kg)	100.00	100.00	100.00	100.00
Moisture content (%w.b.)	10.14	8.25	8.18	7.41
Price (THB/kg)	13.32	14.2	12.45	21
Reduce cost (%)	36.58	33.25	40.71	-

Note: * is the commercial feed used as control. THB refers to Thai Baht; 1 USD was approximately equivalent to 33 THB at the date of writing.

## Data Availability

Data will be made available on request.
